# Conservative Treatment of Sesamoiditis: A Systematic Literature Review with Individual-Level Pooled Data Analysis

**DOI:** 10.3390/medicina61071215

**Published:** 2025-07-03

**Authors:** Carlo Biz, Maria Chiara Maccarone, Valentina Bonso, Elisa Belluzzi, Stefano Masiero, Nicola Luigi Bragazzi, Pietro Ruggieri

**Affiliations:** 1 Orthopedics and Orthopedic Oncology, Department of Surgery, Oncology and Gastroenterology (DiSCOG), University-Hospital of Padova, Via Giustiniani 3, 35128 Padova, Italy; carlo.biz@unipd.it (C.B.); pietro.ruggieri@unipd.it (P.R.); 2 Centre for Mechanics of Biological Materials, University of Padova, 35131 Padova, Italy; 3 Rehabilitation Unit, Department of Neuroscience, University of Padova, 35128 Padova, Italy; mariachiara.maccarone@phd.unipd.it (M.C.M.); stefano.masiero@unipd.it (S.M.); 4 Department of Neuroscience, University of Padova, 35128 Padova, Italy; valentina.bonso@studenti.unipd.it; 5 Musculoskeletal Pathology and Oncology Laboratory, Department of Surgery, Oncology and Gastroenterology (DiSCOG), University of Padova, Via Giustiniani 3, 35128 Padova, Italy; 6 Physical Medicine and Rehabilitation School, Department of Neuroscience, University of Padova, 35128 Padova, Italy; 7 Laboratory for Industrial and Applied Mathematics (LIAM), Department of Mathematics and Statistics, York University, Toronto, ON M3J 1P3, Canada; robertobragazzi@gmail.com

**Keywords:** sesamoiditis, conservative treatment, systematic review, return to sport

## Abstract

*Background and Objectives*: Sesamoiditis is a painful and functionally limiting condition that affects the sesamoid bones of the hallux, frequently seen in athletic populations. Despite its clinical relevance, there are no standardised guidelines for its conservative management. This systematic review aims to evaluate the effectiveness of conservative treatments for sesamoiditis by summarising individual-level data from published studies. *Materials and Methods*: A comprehensive literature search was conducted in PubMed/MEDLINE, Scopus, ISI/Web of Science, and PEDro (Physiotherapy Evidence Database) up to December 2024 in accordance with PRISMA 2020 guidelines and following a protocol specifically devised for rare or underrepresented medical conditions. Eligible studies included case reports and case series involving patients aged ≥16 years who were conservatively treated for clinically and/or radiologically diagnosed sesamoiditis. Data on patient demographics, diagnosis, type and duration of treatment, pain- (Visual Analogue Scale (VAS) and Numeric Rating Scale (NRS)) and function-related (Foot and Ankle Ability Measure (FAAM) and Foot and Ankle Outcome Score (FAOS)) outcomes, and return to activity were extracted. Study quality was assessed using Joanna Briggs Institute (JBI) critical appraisal tools. Pooled effect sizes were computed where applicable. *Results*: Out of 2380 initial records, 11 studies comprising 59 patients (29 females) were included. Treatments varied widely, including orthotics, corticosteroid injections, physical therapy, and biologic approaches such as concentrated bone marrow aspirate (CBMA). VAS scores improved in 66% of cases. NRS scores returned to baseline in some patients after initial improvement, revealing recurrence. FAAM and FAOS subscales showed functional improvements, particularly in sports-specific domains. Return to activity varied: in a case series, 45.4% resumed pain-free sports participation, while others remained symptomatic. *Conclusions*: Conservative treatment options for sesamoiditis showed variable effectiveness with promising outcomes in selected patients. Corticosteroid injections and orthotics appeared beneficial, but high recurrence and limited functional recovery persisted in some cases. Standardised treatment protocols and high-quality prospective studies are needed to improve clinical decision-making and optimise non-surgical management.

## 1. Introduction

“Sesamoiditis” is a commonly used term in clinical practice, yet it lacks a universally accepted definition in the medical literature. It generally refers to a painful and often debilitating musculoskeletal condition characterised by the acute or chronic inflammation of the sesamoid bones of the hallux and the surrounding soft tissues [1,2]. Among these, the medial (tibial) sesamoid is more frequently affected due to its larger size and its primary role in bearing weight [3].

The sesamoid bones protect tendons, reduce mechanical stress during joint movement, and optimise biomechanical efficiency. Additionally, they act as shock absorbers at the first metatarsophalangeal (MTP) joint, transmitting between 50% and 300% of body weight, depending on the type of movement and applied load [4].

Beyond inflammation, the term “sesamoiditis” also includes osteochondral lesions, conditions that may result from various causes affecting the cartilage and subchondral bone [5]. Due to their relatively poor blood supply, sesamoids are also particularly vulnerable to necrosis [6].

Sesamoiditis is frequently observed in athletes, with an incidence of 4.2%, accounting for 2.2% of all lower limb injuries and 18.3% of injuries involving the first MTP joint [2,7]. Overall, sesamoid injuries constitute approximately 9% of all injuries affecting the ankle-foot complex [8].

While the condition may arise in the absence of trauma [6], common mechanisms of injury include the acute dorsiflexion of the first MTP joint and repetitive stress, such as those associated with running and jumping [1]. Sports like soccer, tennis, basketball, and dance have been identified as high-risk activities [9]. Additional risk factors include prior foot conditions (e.g., turf toe or hallux rigidus), trauma to the hallux, excessive foot pronation, and inappropriate footwear [2,9].

Clinically, sesamoiditis presents with localised pain beneath the first metatarsal head or more diffuse discomfort across the plantar surface of the hallux, often worsening with movement and weight-bearing. In cases of direct trauma, oedema and ecchymosis may also be present [1]. This condition can significantly impact gait and functional mobility, leading to compensatory movement patterns that may be disabling for both athletes and less active individuals [2,7].

Diagnosis primarily relies on clinical evaluation [2,7], including the execution of specific manoeuvres such as the Passive Axial Compression (PAC) test, which reproduces pain associated with the sesamoids [10]. Imaging modalities such as X-rays, computed tomography (CT), and magnetic resonance imaging (MRI) are frequently employed to confirm the diagnosis and exclude alternative pathologies [3,8,11,12,13].

Treatment for sesamoiditis typically begins with conservative management strategies aimed at alleviating pain, reducing inflammation, and promoting healing [2]. These approaches include activity modification, orthotic interventions, physical therapy, pharmacological treatments, and, in some cases, corticosteroid injections. Activity modification involves avoiding activities that put excessive pressure on the affected area, such as running, jumping, or wearing high heels. This allows the inflamed sesamoid bones to rest and recover. Orthotic interventions (such as custom insoles or foot padding) help offload pressure from the sesamoids and redistribute weight more evenly across the foot, relieving relief from pain and preventing further irritation. Physical therapy techniques (such as shockwave therapy and laser therapy) aim to reduce inflammation and stimulate tissue repair. Shockwave therapy uses sound waves to promote healing in the affected area, while laser therapy uses light energy to accelerate recovery and alleviate pain. Pharmacological treatments, primarily nonsteroidal anti-inflammatory drugs (NSAIDs), are used to manage pain and inflammation. Depending on the severity of symptoms, topical or oral NSAIDs may be prescribed. In cases where conservative treatments fail to provide relief, corticosteroid injections may be considered [9]. These injections deliver anti-inflammatory medications directly to the affected area, offering rapid pain relief and reducing inflammation. While these conservative treatments are often effective, their success rates vary, and surgical intervention may be required in refractory cases [2,3,4,7,9,12,14,15].

Despite the growing interest in sesamoid-related diseases, there is a lack of comprehensive studies systematically evaluating and comparing conservative treatment options for sesamoiditis. Consequently, evidence-based guidelines for its management are still limited [2,7]. This systematic review aims to identify and analyse all conservative treatment strategies described in the literature for managing sesamoiditis. The goal is to explore therapeutic options that may prevent or delay the need for surgery and, where possible, compare their effectiveness to determine the most optimal management strategies.

## 2. Materials and Methods

### 2.1. Search Strategy

A systematic review was conducted following the protocol proposed by Nambiema et al. (2021) [16] based on the PRISMA guidelines (Preferred Reporting Items for Systematic Reviews and Meta-Analyses) [17]. This approach outlined the methodology to be adopted for systematic reviews, which, when the topic was rare or underrepresented in the literature, also included case reports and case series studies. Both guidelines were employed to monitor the research process and to draft the document itself. The study protocol has been registered within the Open Science Framework repository (Identifier code: DOI:10.17605/OSF.IO/KMURE).

A comprehensive literature search was conducted across four major scholarly electronic databases: PubMed/MEDLINE, ISI/Web of Science (WoS), Scopus, and PEDro (Physiotherapy Evidence Database), with no temporal restrictions. An advanced search was performed in each database using the following combined keywords: “sesamoid”, “conservative”, “therapy”, “rehabilitation”, “treatment”, “exercise”, “human”, and their respective Boolean operators. The full search strings used in each database are provided in Table 1. All databases were consulted from inception until December 2024.

### 2.2. Inclusion Criteria

The search was limited to articles written in English and classified as original articles. Retrospective studies, prospective investigations, randomised controlled studies (RCTs), case series, and case reports were selected if they met the following PICO criteria:

- Population/patients: Individuals diagnosed with sesamoiditis (based on clinical and imaging findings), including athletes (amateur, semi-professional, professional, and elite) and non-athletes, of either sex/gender, aged 16 years or older.
- Intervention: Implementation of conservative treatments for sesamoiditis, including physiotherapy, physical therapies (such as shockwave or laser therapy), corticosteroid injections, and/or orthotics.
- Comparator: Studies were selected if they included pre- and post-intervention measurements.
- Outcome: Studies reporting outcomes such as pain reduction at rest or during activity, improvement in quality of life, and return to activity or sport were included. Studies using the following validated scales or questionnaires were deemed eligible: the Visual Analogue Scale (VAS) and the Numeric Rating Scale (NRS) for pain-related outcomes; the Foot and Ankle Ability Measure (FAAM) divided into two subscales, i.e., FAAM-Sport and FAAM-ADL, for function-related outcomes and to evaluate functional capacity in sports and activities of daily living (ADL) for individuals with foot or ankle problems; and the Foot and Ankle Outcome Score (FAOS), used to assess clinical outcomes (symptoms, pain, ADL, sports and leisure activities, and quality of life) related to foot and ankle disorders.

### 2.3. Exclusion Criteria

All studies analysing populations under the age of 16 were excluded. Studies involving patients with fractures, osteochondromas, flexor hallucis longus tenosynovitis, sesamoid bone dislocations, or other foot conditions (e.g., hallux valgus, hallux rigidus, or turf toe) were also excluded. Studies in which all participants underwent surgical treatment were excluded, whereas no restrictions were imposed on studies that included at least one subject treated conservatively. Articles not written in English were excluded. Additionally, the following types of studies were excluded: narrative reviews, systematic reviews, meta-analyses, interviews, book chapters, opinions, comments/commentaries, technical notes, letters, editorials, or articles for which the full text was unavailable. Although reviews were not retained for inclusion, their reference lists were scanned to increase the likelihood of identifying additional relevant studies.

### 2.4. Study Selection

As previously mentioned, this procedure was based on the PRISMA-2020 guidelines [17] and the protocol proposed by Nambiema et al. (2021) [16], which involved identifying and evaluating studies suitable for the review for rare or under-represented medical conditions. After removing duplicates from the initial database search, two independent reviewers (VB and MCM) carried out the screening by examining the title and/or abstract of all articles. When it was impossible to determine the inclusion or exclusion of a specific article based solely on the title or abstract, the full text was retrieved and analysed. Any disagreements between the reviewers were resolved through discussion, with consultation from a third reviewer (CB) if necessary. The inter-rater agreement was high (Cohen’s kappa > 0.90). An open-source software (Zotero version 6.0.37 for Mac, Corporation for Digital Scholarship) was used for both citation management and duplicate removal.

### 2.5. Data Extraction

The most important data from the included studies were systematically extracted and recorded in an ad hoc Excel file. The extracted information included the authors, publication date, study design, participant groups, number of patients, sex/gender, age, number of patients lost to follow-up, duration of follow-up, number and type of sesamoids, time between diagnosis and start of treatment, duration of treatment, and various outcome measures related to pain (VAS and NRS), function (FAAM-Sport, FAAM-ADL, and FAOS), and return to activity. Two reviewers (VB and MCM) independently performed the data extraction. Any discrepancies were resolved through discussion and, when necessary, with the assistance of a third reviewer (CB). The inter-rater agreement was high (Cohen’s kappa > 0.90).

To address missing data, data extraction was limited to commonly reported variables.

### 2.6. Quality Assessment

To address the variability in study design and methodology among the included studies, the Joanna Briggs Institute (JBI) critical appraisal tools were used to systematically evaluate their quality [18].

These tools provide customised checklists tailored to different study types, enabling a structured assessment of various methodological aspects. Each item on the checklist is rated using one of the following options: “yes”, “no”, “unclear”, or “not applicable (N/A)”. These tools are based on core methodological principles that assess the transparency, completeness, and reliability of reporting. For instance, in the appraisal of case reports, the checklist evaluates whether essential clinical details—such as patient demographics, history, diagnostic procedures, interventions, and outcomes—are clearly and systematically described. For case series, the tool focuses on consistency in inclusion criteria, reliability of condition measurement, completeness of reporting, and appropriateness of statistical analysis [19]. This structured approach helps ensure that the included studies meet minimum standards of methodological rigour, allowing for a more trustworthy summary of evidence.

The PEDro scale was not applicable for quality assessment, as none of the included studies were clinical trials.

### 2.7. Statistical Analysis and Individual-Level Data Pooled Analysis

Given the nature and heterogeneity of the available data, an individual-level pooled data analysis was conducted, primarily using descriptive statistical methods. Continuous variables (such as age, duration of symptoms, VAS/NRS scores, and functional outcome measures) were summarised using means, standard deviations (SDs), medians and ranges, where appropriate. Categorical variables (such as sex/gender, laterality, athletic status, treatment type, and return to sports) were reported as frequencies and percentages. Data processing and tabulation were performed using the commercial software “Statistical Package for the Social Sciences” (SPSS version 28, IBM, Armonk, NY, USA).

## 3. Results

### 3.1. Literature Search

**Figure 1:**
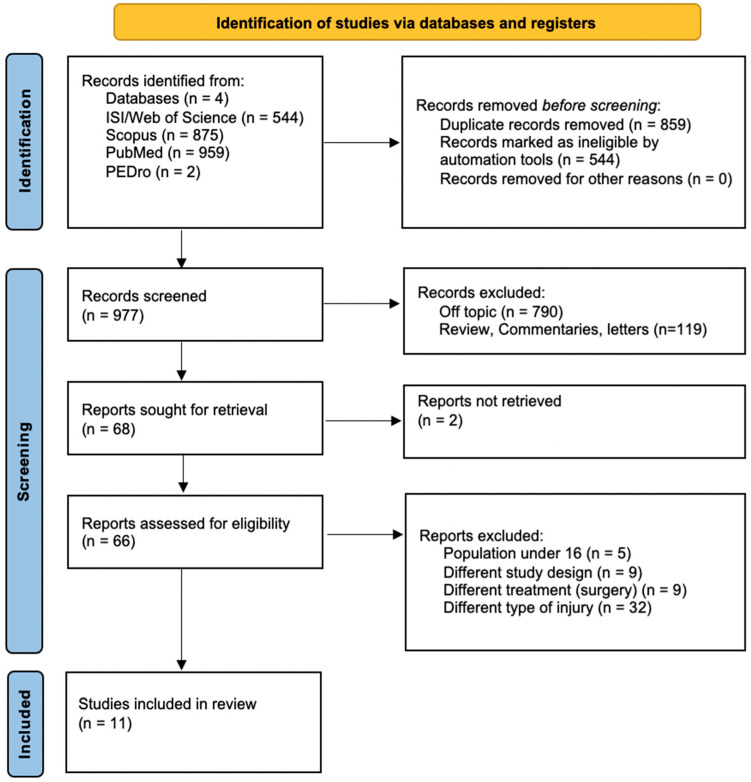
Flowchart of the search conducted in compliance with the criteria outlined in the “Preferred Reporting Items for Systematic Reviews and Meta-Analyses” (PRISMA) guidelines.

Initially, 2380 records were identified across the four databases: ISI/Web of Science (*n* = 544), Scopus (*n* = 875), PubMed/MEDLINE (*n* = 959), and PEDro (*n* = 2). Before screening, 859 duplicates were removed using Zotero reference manager software and 544 results were automatically excluded as not eligible by automation tools. During the screening phase, 977 records were examined. Of these, 790 were excluded for being off-topic, and 119 were identified as reviews, commentaries, or letters. Additionally, 2 records were reported as not retrievable, and 66 were flagged for retrieval. Upon the assessment of 66 full-text articles for eligibility, 55 were excluded for the following reasons: population under 16 years of age (*n* = 5), inappropriate study design (*n* = 9), focus on surgical treatment (*n* = 9), and medical conditions other than sesamoiditis (*n* = 32). Ultimately, 11 studies met all inclusion criteria and were incorporated into the final review. The selection process is depicted in Figure 1.

### 3.2. Demographics of the Studies Included

The reviewed literature consisted of seven case reports [20,21,22,23,24,25,26] and four case series [27,28,29,30], of which three were retrospective and one was prospective. Sample sizes ranged from single-patient case reports [20,21,22,23,24,25,26] to series involving up to 27 individuals [29], for a total of 59 subjects. The proportion of female participants varied across studies; some exclusively involved only female subjects, while others included mixed sex/gender distributions. Overall, 29 of the 59 participants were female, representing 49.2% of the pooled sample.

Participant ages spanned a wide range, from adolescent [30] to older adult populations [29], with an average age of 34 years. Body mass index (BMI) was reported in only a limited number of studies. Choi et al. reported a mean BMI of 24.5 ± 2.4 kg/m^2^ [29], while Frey and Koehle documented a BMI of 24.9 kg/m^2^ for their single case [26]. Athletic involvement was common in most cases (53 out of 59, 89.8%), ranging from recreational to professional levels. Axe et al. and Shimozono et al. included participants engaged in diverse sports activities such as football, dance, shot put, and tennis [28,30]. Choi et al. included the most heterogeneous sample in terms of sports, with participants involved in running, golf, weightlifting, futsal, and sports dancing [29]. Follow-up periods, when reported, varied considerably from short-term follow-up (Shin et al., with a 1-day follow-up) [24] to long-term monitoring (Shimozono et al., with a follow-up of up to 34 months) [30]. Further details are reported in Table 2.

### 3.3. Conditions

A total of 60 feet were treated, with an equal distribution between the right (*n* = 30) and left (*n* = 30) sides. In total, 62 sesamoid bones were managed conservatively, of which 51 (82.3%) were medial and 11 (17.7%) were lateral. The duration of symptoms ranged from 1 week [28]–10 days [27] to 5 years [22].

A broad spectrum of sesamoid disorders was described, encompassing both acute and chronic pathologies. Although terminology varied slightly across studies, the conditions can be categorised into six main diagnostic entities, reflecting the anatomical location and underlying pathophysiology. The most frequently reported condition was sesamoiditis, identified in four studies: Frey and Koehle, Kumar et al., Benslima et al., and Thompson et al. [21,22,25,26], with the latter also noting coexisting osteonecrosis. The isolated osteonecrosis of the sesamoid bones was reported in three studies. Aşkın et al. and Callahan et al. described cases of isolated sesamoid osteonecrosis confirmed by clinical imaging [20,27]. Maganinho et al. presented a case of subhallucal sesamoid osteonecrosis [23]. Atraumatic medial sesamoid pain (MSP) was defined by Choi et al. [29]. Generalised sesamoid pain was discussed in Axe et al. [28]. Sesamoid hallux disorders were classified under a single umbrella term in the study by Shimozono et al. [30]. Finally, symptomatic hallucal interphalangeal sesamoid bones were reported in the study by Shin et al. [24].

### 3.4. Treatment Protocols

Conservative treatment approaches were heterogeneous across the included studies, with protocols generally combining rest, mechanical offloading, pharmacologic therapy, physical modalities, and, in selected cases, injections or biologics.

Rest, ranging from complete rest to activity limitation/modification and including non-weight-bearing (NWB) protocols, was documented in five studies. In Aşkın et al. (2017), one of the two patients was managed with rest [27]. Benslima et al. prescribed a three-month period of rest during which only low-impact activities such as swimming were allowed [21]. Similarly, Choi et al. included activity restriction in their initial conservative phase [29]. Shimozono et al. and Callahan et al. incorporated an NWB period [20,30].

The use of orthotics, in-shoe padding or immobilisation devices was reported in six studies. Aşkın et al. provided an orthopaedic insole, and a short leg cast as part of the conservative protocol [27]. Axe and Ray used specific orthotic devices tailored to offload the sesamoid [28]. Choi et al. prescribed stiffened offloading insoles and boot walker application with crutches [29]. Kumar et al. used soft in-shoe pads to relieve local pressure [22]. Shimozono et al. included customised orthotics during the post-intervention rehabilitation period [30], and Callahan et al. included axillary crutches [20].

Physical therapy was described in two studies. Aşkın et al. implemented a structured rehabilitation programme that included contrast bath therapy, range-of-motion exercises, progressive resistance training, and balance and coordination activities [27]. Similarly, Callahan et al. incorporated physical therapy in their treatment protocol [20].

NSAIDs or oral analgesics were prescribed in three studies. Benslima et al. used NSAIDs [21], while Choi et al. and Kumar et al. administered oral analgesics as part of the conservative treatment protocol [22,29].

Shockwave therapy and transcutaneous electrical nerve stimulation (TENS) were each documented in one study: Thompson et al. and Aşkın et al. [25,27], respectively.

Corticosteroid injections were reported in five studies [22,23,24,26,29]. Frey and Koehle administered two local corticosteroid injections [26]. Similarly, Choi et al. included corticosteroid injections, with some patients receiving up to three ultrasound-guided injections at three-week intervals [29]. Kumar et al. used a single ultrasound-guided local injection [22]. Maganinho et al. performed an image-guided corticosteroid injection consisting of triamcinolone acetonide and lidocaine into the space between the sesamoid bone and the flexor hallucis longus tendon [23]. Shin et al. reported the use of ultrasound-guided injections for the symptomatic interphalangeal sesamoid bones of the hallux [24].

In addition, biologic therapies were used in isolated cases. Callahan et al. (2025) described a single leukocyte-rich platelet-rich plasma (PRP) injection under ultrasound guidance [20], while Shimozono et al. reported the use of CMBA [30].

The duration of treatment varied from 1 day (Shin et al. [24] and Maganinho et al. [23]) to 22 months (Axe and Ray) [28]. Finally, the time from diagnosis to treatment initiation ranged from 0 days [21,22,23,24,27,29] to a delay of up to 10 months [26]. Further details are reported in Table 3.

### 3.5. Outcomes

#### 3.5.1. Pain-Related Outcomes

##### VAS

Data on VAS scores were available from three studies [25,29,30], encompassing a total of 41 participants. In the study by Choi et al., the baseline VAS was 8.3 (range: 6–10) [29]. After three months of conservative treatment, 14 subjects (51.9%) continued to experience persistent pain, with a VAS of 6.7 (range: 5–9), while 13 subjects (48.1%) showed improvement, reporting a reduced VAS of 2.3 (range: 1–5). Shimozono et al. reported a significant reduction in VAS scores from 5.8 (range: 4–8) to 1.5 (range: 0–4) following treatment [30]. Thompson et al. documented a baseline VAS of 6, which decreased to 2 at the end of treatment and reached 0 at the one-year follow-up. Overall, a reduction in VAS scores was observed in 66% of the cases, indicating clinical improvement in two-thirds of the patients [25]. Further details are reported in Table 4.

##### NRS

NRS scores were reported in two studies (Table 4). Callahan et al. documented an initial NRS score of 4, which improved to 0 after 2 months but returned to 4 at the 2-year follow-up, indicating initial resolution followed by long-term recurrence [20]. Shin et al. reported a higher baseline NRS of 8, which decreased to 0 following treatment [24].

#### 3.5.2. Function-Related Outcomes

##### FAAM

Information concerning FAAM scores was reported only by Choi et al., who documented a baseline FAAM-ADL score of 51.9 (range: 42–63) [29]. This study provided data stratified into two groups based on the persistence of pain six months after treatment. In the group experiencing persistent pain, this value slightly increased to 52.1 (range: 42–64), while it significantly increased to 73.4 (range: 68–79) in the group without persistent pain. Similarly, the FAAM-Sports score at baseline was 14.7 (range: 12–21). In the persistent pain group, the score remained low at 14.3 (range: 11–22), while in the pain-free group, it significantly increased to 26.6 (range: 23–32), reflecting a better functional outcome (Table 5).

##### FAOS

Only Shimozono et al. assessed clinical outcomes using the FAOS [30]. At baseline, the mean FAOS subscale scores were as follows: symptoms, 64.0 (±7.4); pain, 56.1 (±11.4); daily activities, 68.9 (±8.6); sports activities, 36.8 (±9.4); quality of life, 30.5 (±8.2); and overall score, 51.3 (±5.6). After treatment, all subscales showed substantial improvement: symptoms increased to 90.8 (±7.5), pain to 83.8 (±17.4), daily activities to 92.8 (±7.3), sports activities to 72.5 (±20.1), quality of life to 64.6 (±22.7), and overall score to 81.4 (±13.4) (Table 5).

#### 3.5.3. Return to Activity

Return to activity was reported in six studies, with considerable variability depending on treatment and baseline patient characteristics. Axe and Ray provided a detailed account involving different patient profiles [28]. Among five varsity athletes, three experienced immediate and almost complete symptom relief with orthoses, one achieved gradual resolution over four weeks, and one required six weeks for pain resolution. In contrast, patients who did not use orthoses continued to experience symptoms—three during basketball activities and one during ADLs; all three refused surgical intervention. Two recreational runners also remained symptomatic without orthotic support and continued using them prophylactically. One of these individuals reported persistent symptoms during aerobics, while the other experienced discomfort when wearing heels; both declined surgery. Additionally, a musician became asymptomatic immediately after initiating orthotic use, eventually achieving symptom resolution even without orthoses. Two further patients, including a professional dancer and a jogger, underwent the surgical excision of the sesamoid and returned to full activity within three months postoperatively—the dancer to ballet and the jogger to extended running distances.

Callahan et al. described a case of a ballet dancer who resumed regular auditions three months following intervention [20]. Choi et al. provided data stratified into two groups based on the persistence of pain six months after treatment [29]. In the group with persistent pain (*n* = 12), no patients were able to return to sports without pain. Three patients (25%) could participate in sports but experienced mild post-sports musculoskeletal pain (MSP). Five (41.7%) were able to perform daily activities but could not return to sports due to MSP, and four (33.3%) continued to experience residual MSP even during ADLs. In contrast, in the group without persistent pain (*n* = 11), five patients (45.4%) returned to their previous level of sports participation without pain, four (36.4%) resumed sports with only mild MSP, and two (18.2%) remained limited to daily activities due to pain. None of the patients in this group experienced residual MSP during ADLs. Frey and Koehle documented a case in which the patient returned to running three months post-treatment and completed a 10 km race in 75 min at four months, with only minimal residual discomfort [26]. Shimozono et al. reported that 8 of 11 patients (73%) engaged in sports before undergoing CBMA injection returned to athletic activity, with 7 regaining their pre-injury performance level [30]. The remaining three patients (27%) required additional treatment. Thompson et al. described a patient who resumed tennis six weeks after completing radial extracorporeal shockwave therapy (rESWT) [25]. Lastly, Shin et al. noted a rapid resolution of symptoms, with complete pain relief reported the day following treatment and the patient resuming normal daily activities, including shopping, immediately thereafter [24]. Further details are reported in Table 6.

### 3.6. Quality Assessment

Among the case series studies, all four studies [27,28,29,30] demonstrated generally strong methodological quality (Table 7). Most items were rated as “Yes”, particularly in domains related to participant inclusion (Q1–Q3), outcome assessment (Q6–Q8), and statistical reporting (Q9). However, Q4 (consecutive inclusion of participants) was rated “No” or “N/A” in three out of four case series, reflecting possible selection bias due to the lack of clearly defined enrolment protocols. Additionally, both Shimozono et al. [30] and Choi et al. [29] failed to fulfil Q5, which pertains to the completeness of demographic and clinical information, potentially limiting external validity. The methodological quality of the case reports was more heterogeneous but remained generally acceptable. All seven included case reports [20,21,22,23,24,25,26] met criteria for clear case description (Q1–Q3), intervention detail (Q4–Q5), and outcome clarity (Q8). Items Q6 and Q7, which evaluate post-intervention follow-up and adverse events reporting, were consistently marked as “N/A” due to the inherent limitations of the case report format or omission in reporting.

## 4. Discussion

This systematic review offers the first comprehensive summary of individual-level data on conservative treatments for sesamoiditis, a musculoskeletal condition that, despite its potential to significantly impair function and quality of life, remains understudied in both clinical practice and research. The included studies, designed as case reports and case series, mostly retrospective with the single exception of a prospective investigation, highlight the diversity of conservative therapeutic approaches and underscore the lack of standardised treatment protocols.

The findings demonstrate that conservative interventions, including orthotic therapy, corticosteroid injections, physical rehabilitation, and biological approaches such as CBMA, can yield meaningful symptom relief and functional recovery in selected patients. In particular, interventions that incorporated image-guided corticosteroid injection, orthotics, and structured rehabilitation [20,30] emerged as the most frequently adopted and clinically beneficial interventions among the studies, allowing patients to return to sports or daily activities with reduced pain.

However, the outcomes were heterogeneous, with several studies reporting persistent symptoms or symptom recurrence, even after initial improvement. This variability may be attributable to differences in patient characteristics (e.g., BMI, age, type of sport), diagnostic accuracy, or the timing and type of intervention, although not all variables were consistently reported across studies. The study by Choi et al. notably showed that younger age, higher BMI, and the presence of a bipartite sesamoid were significant predictors of poor response to conservative therapy [29]. Moreover, the observation that MRI findings did not consistently correlate with symptom severity raises important considerations regarding the underlying mechanisms of pain, which may not be solely structural but may involve neuromechanical or inflammatory pathways. However, soft tissue signal changes were the most commonly reported findings with MRI, followed by intraosseous abnormalities and chondral/subchondral lesions. The authors concluded that conservative management is often insufficient and that MRI may help guide future treatment in refractory cases.

The inconsistency in imaging findings, treatment modalities, and outcome reporting across the included literature further complicates the ability to draw firm conclusions or generate clinical guidelines. While some cases demonstrated sustained symptom remission and return to high-level athletic function [25,30], others documented relapse or incomplete recovery, suggesting that conservative treatment may serve as a delaying rather than curative strategy in specific individuals [20,29].

Although the primary focus of this review was on conservative management, surgical intervention remains a consideration in refractory cases, particularly when pain persists despite adequate non-operative treatment. Surgical options, such as partial or complete sesamoidectomy, have been reported in the literature with variable outcomes. Studies have demonstrated that sesamoidectomy can lead to significant pain relief and high rates of return to activity. For instance, a systematic review reported that 94.4% of patients returned to sports, with 90.0% returning to their previous level, at a mean of 11.8 weeks postoperatively. Overall complication rate was 22.5% and the revision rate was 3.0% [31]. Similarly, a two-centre study focusing on athletes found an average return to activity time of 11.1 weeks, with a low complication rate of 5.7% [32].

However, potential complications such as hallux valgus, diminished push-off strength, and altered gait mechanics have been reported. Moreover, there is a limited consensus on surgical indications, techniques, and rehabilitation protocols. Given these concerns, surgery should be reserved for well-selected patients after a multidisciplinary evaluation and comprehensive conservative management [31].

A noteworthy limitation of the current evidence on conservative treatment is the predominance of low-level study designs, with small sample sizes and limited follow-up durations. Moreover, although some studies used VAS, NRS, FAAM, or FAOS, the lack of standardised outcomes measures reduces data comparability and limits the possibility of more comprehensive pooled data analysis. Despite these limitations, this review identifies potentially promising therapeutic pathways that should be explored in future research to contribute to clinical practice.

In conclusion, the conservative management of sesamoiditis can be effective, particularly when initiated early and tailored to the patient’s specific clinical profile. However, the observed recurrence and persistent functional impairment in some cases emphasise the need for further prospective, high-quality studies to validate the most effective treatment strategies and to elucidate the pathophysiological mechanisms underlying sesamoid-related pain. The development of standardised diagnostic and therapeutic pathways remains a priority to optimise patient outcomes and reduce unnecessary surgical interventions.

### 4.1. Strengths and Limitations

A notable strength of this review lies in its rigorous adherence to PRISMA 2020 guidelines and the inclusion of diverse data sources, thereby maximising the breadth of available evidence in a field where randomised trials are currently lacking. Even with small samples, individual-level pooled data analysis enables a more nuanced understanding of treatment responsiveness across patient subgroups. However, several limitations must be acknowledged. First, the inclusion of case reports and case series, though necessary due to the rarity of the condition, introduces inherent risks of selection and publication biases, as well as inconsistent reporting standards. This may lead to an overestimation of treatment efficacy, especially in the absence of control groups or standardised outcome assessments. Second, the heterogeneity in follow-up duration, outcome measures, and intervention protocols reduces comparability between studies and precludes more statistically robust meta-analytic techniques. The use of non-consecutive sampling and the short or undefined follow-up periods in several reports may further compromise the generalizability and long-term applicability of the observed outcomes. Third, there was minimal reporting on long-term adverse effects or quality-of-life outcomes, essential for assessing the sustainability of conservative approaches. Finally, return-to-sports criteria were inconsistently defined, with a few studies applying standardised functional benchmarks.

### 4.2. Future Directions

Future research should prioritise the development of prospective multicentre registries and randomised controlled trials that compare conservative modalities directly with one another and with surgical treatments. Standardised diagnostic criteria, imaging protocols, and outcome metrics, including clinician-reported and patient-reported outcomes, are urgently needed. Further, exploring biological and biomechanical predictors of treatment success (e.g., bipartite sesamoids, foot biomechanics, tissue perfusion) may enhance the stratification and personalisation of care. The role of novel interventions such as orthobiologics (e.g., PRP, CBMA) and shockwave therapy warrants further evaluation through high-quality trials. While the conservative treatment of sesamoiditis offers a viable first-line approach, its efficacy remains inconsistent across populations and sports profiles. A paradigm shift toward evidence-based, patient-tailored treatment algorithms, grounded in prospective data and guided by imaging and clinical phenotyping, will be critical for advancing the management of this challenging condition.

## 5. Conclusions

This systematic review demonstrates that conservative interventions for sesamoiditis yield variable outcomes. Approximately two-thirds of patients achieve substantial pain relief, yet symptom recurrence and incomplete functional recovery are common. Only about half of athletic patients fully return to sports without pain, underscoring the limitations of non-surgical care in high-impact activities. In non-athletic individuals, conservative management alleviates pain and improves daily function, although residual symptoms may persist. Orthotic offloading and corticosteroid injections are the most consistently beneficial interventions, providing significant short-term relief. Physical therapy modalities (such as structured rehabilitation exercises and shockwave therapy) restore function. Despite limited evidence, emerging biologic therapies (e.g., PRP or CBMA injections) also show promise in recalcitrant cases. Clinically, these findings support a stepwise, multimodal conservative approach tailored to patient needs. Initial management should combine activity modification, NWB/weight-bearing offloading with appropriate orthoses or footwear changes, and analgesics (e.g., NSAIDs) for pain control. If symptoms persist, the judicious use of image-guided corticosteroid injections is advised. In athletic patients, extended rehabilitation and careful monitoring are crucial before resuming sports. Ultimately, the heterogeneity of current evidence highlights the need for standardised treatment protocols and high-quality prospective studies to establish definitive guidelines and optimise the non-operative management of sesamoiditis.

## Figures and Tables

**Table 1:** Search strategies used across four major scholarly electronic databases for retrieving studies on the conservative management of human sesamoid conditions.

Database	Search Strategy
Scopus	TITLE-ABS-KEY (sesamoid*) AND TITLE-ABS-KEY (human) AND TITLE-ABS-KEY (conservative OR therapy OR rehabilitation OR treatment OR exercise)
PubMed/MEDLINE	(sesamoid*) AND (conservative OR therapy OR rehabilitation OR treatment OR exercise) AND human
ISI/Web of Science (WoS)	sesamoid* (Topic) AND (conservative OR therapy OR rehabilitation OR treatment OR exercise) (Topic)
PEDro	Abstract and Title: sesamoid*

**Table 2:** Characteristics of the studies included in the present review.

Study	Study Design	Sample Size (Female/Male)	Age	Athletic Subjects	Follow-Up	Patients Excluded/Lost to Follow-Up
Axe and Ray, 1988 [28]	Retrospective case series	10 (6/4)	22.7 ± 4 (median 21.5 [17–30])	10 (college-level football player, *n* = 4; college-level shot putter, *n* = 1; professional dancer, *n* = 1; recreational athletics, *n* = 2; scholastic marching band member, *n* = 1; distance jogger, *n* = 1)	19.7 months [9–24]	0
Frey & Koehle, 2013 [26]	Case report	1 (1/0)	63	1 (recreational runner)	4 months	0
Shin et al., 2013 [24]	Case report	1 (1/0)	29	0	1 day	0
Kumar et al., 2015 [22]	Case report	1 (0/1)	49	0 (bus driver)	Lost to follow-up	Lost to follow-up
Aşkın et al., 2017 [27]	Retrospective case series	2 (2/0)	31 ± 1.4 [30–32]	0	1.5 ± 0.7 months [1,2]	0
Thompson et al., 2017 [25]	Case report	1 (0/1)	52	1 (recreational club tennis player)	1 year	0
Shimozono et al., 2022 [30]	Retrospective case series	13 (10/3) out of 15 consecutive patients initially included	26.9 [16–39]	11/13 (professional level, *n* = 4; college level, *n* = 3; recreational level, *n* = 4)	20.1 months [12–34]	0
Benslima et al., 2023 [21]	Case report	1 (1/0)	30	1 (recreational runner)	1 month	0
Callahan et al., 2024 [20]	Case report	1 (1/0)	28	1 (professional musical theatre performer, dancing ballet and hip hop)	2 years	0
Choi et al., 2024 [29]	Prospective case series	27 (6/21)	40 ± 11.5 [24–67]	27 (running, *n* = 8; golf, *n* = 5, weightlifting with squatting, *n* = 5; sports dancing, *n* = 5; futsal, *n* = 4)	6 months	9 (4 lost after initial treatment, 2 after injection, 3 after brace application with crutches)
Maganinho et al., 2024 [23]	Case report	1 (1/0)	22	1 (professional dancer or “ballerina”)	3 months	0

**Table 3:** Laterality, type and number of sesamoids treated, clinical condition, symptoms duration before treatment, treatment modalities, duration of treatment, and time elapsed from diagnosis to initiation of treatment in the included studies.

Study	Number of Feet Treated (Right/Left)	Total Sesamoids (Medial/Lateral)	Condition	Duration of Symptoms	Treatment	Duration of Treatment	Time Between Diagnosis and Start of Treatment
Axe and Ray, 1988 [28]	10 (6/4)	10 (6/4)	Sesamoid pain (“a sharp pain beneath the first metatarsal head upon weightbearing”)	From 1 week to >2 years	Custom-fitted orthoses	From 2 to 21 months (full-time); from 4 to 22 months (part-time)	10 days
Frey & Koehle, 2013 [26]	1 (1/0)	1 (1/0)	Sesamoiditis (“pain … notably worse with weight bearing and aggravated by certain types of footwear”)	7 months	Two local corticosteroid injections (5 mg dexamethasone and 0.75 cc 1% lidocaine during the first injection and 6mg of dexamethasone in 1 cc 1% lidocaine during the second injection)	Two injections interspersed by 1 month	10 months
Shin et al., 2013 [24]	2 (1/1)	4 (2/2)	Symptomatic hallucal interphalangeal sesamoid bones (“lower-extremity pain …, which was in the toes, … described as continuous, throbbing, crushing, and burning” and walking difficulties)	9 months (3 months postpartum)	One ultrasound-guided injection of 0.125% levobupivacaine mixed with 10 mg of triamcinolone acetonide.	1 day	0 days
Kumar et al., 2015 [22]	1 (1/0)	2 (1/1)	Subhallucal interphalangeal sesamoiditis (pain “aggravated during application of brakes while driving”)	5 years	One ultrasound-guided local injection of 1 mL of 0.125% levo-bupivacaine mixed with 10 mg of triamcinolone acetonide + the patient was advised to use a soft pad within the shoe underneath the symptomatic area, along with oral analgesic for 2 months	2 months (but patient lost to follow-up)	0 days
Aşkın et al., 2017 [27]	2 (2/0)	2 (2/0)	Sesamoid osteonecrosis	From 10 days to 1 month	(1) Short leg cast for 20 days + contrast bath therapy + TENS 20 min/day + exercise (ROM, progressive resistance, and balance/coordination exercise) (2) Rest + orthopaedic insole + contrast bath therapy	1–2 months	0 days
Thompson et al., 2017 [25]	1 (0/1)	1 (0/1)	Sesamoiditis and sesamoid osteonecrosis (pain “aggravated by walking and playing recreational tennis”)	1 year	Eight sessions of radial extracorporeal shock wave therapy	NA	6 weeks
Shimozono et al., 2022 [30]	13 (5/8)	13 (10/3)	Sesamoid hallux disorders (including sesamoiditis, symptomatic bipartite sesamoids, and avascular necrosis)	11.8 months (from 3 months to 3 years)	One concentrated bone marrow aspirate (CMBA) injection, then 2 weeks of non-weight-bearing postinjection, then full weightbearing was permitted as tolerated with customised orthotics for 3 months; return to play granted 3 months post injection	3 months and 2 weeks	≥3 months of failed previous conservative management (restriction of activities, custom orthotics, nonsteroidal anti-inflammatory drugs, physiotherapy, extracorporeal shockwave therapy, and platelet-rich plasma injection)
Benslima et al., 2023 [21]	1 (1/0)	1 (1/0)	Hallucal sesamoiditis (“chronic great toe pain that had been evolving for several years”)	3 weeks	Non-steroidal-anti-inflammatory-drugs for 5 days + 3 months rest period Only low-impact activities (es. swimming) were recommended	1 month	0 days
Callahan et al., 2024 [20]	1(0/1)	1 (0/1)	Sesamoid osteonecrosis (worsening, aching, and stabbing pain)	6 weeks	One Leukocyte-Rich Platelet-Rich Plasma injection under ultrasound guidance + use of axillary crutches and no weight bearing for the next week + physical therapy for the next 2 weeks post-injection	1 month	15 weeks after failure of conservative therapy (12 weeks of physical therapy, oral nonsteroidal anti-inflammatory drugs, and sesamoid offloading with padding + 3 weeks of use of a CAM boot)
Choi et al., 2024 [29]	27 (12/15)	27 (27/0)	Atraumatic medial sesamoid pain	NA	Stepwise conservative treatment: (a) 3 weeks of oral analgesics, activity restriction and wearing stiffened offloading insoles; (b) and (a) ultrasound-guided local corticosteroid injection of triamcinolone (40 mg) mixed with 1% lidocaine (1 mL); (c) ultrasound-guided local corticosteroid injections attempted at 3-week intervals (maximally 3 times); (d) boot walker application with crutches for 3 weeks	3–4.5 months [12–18 weeks]	0 d
Maganinho et al., 2024 [23]	1 (1/0)	1(1/0)	Osteonecrosis of subhallucal sesamoid bone (“mechanical right forefoot pain …, mostly at the tiptoe dancing movements … and, after a few months … present even when walking”)	1 year	One ultrasound-guided injection of 1 mL of 1% lidocaine mixed with 10 mg of triamcinolone acetonide between the sesamoid bone and the flexor hallux longus tendon	1 day	0 days

**Table 4:** Changes in pain intensity before and after treatment across studies evaluating sesamoid pain using Visual Analogue Scale (VAS) or Numeric Rating Scale (NRS).

Study	Scale	Pain Before Treatment	Pain After Treatment
Shin et al. (2013) [24]	NRS	8 of 10	0 of 10
Thompson et al. (2017) [25]	VAS	6 of 10	2 of 10 after finishing the treatment 0 of 10 after 1 year
Shimozono et al. (2022) [30]	VAS	5.8 (range 4–8)	1.5 (range 0–4)
Callahan et al. (2024) [20]	NRS	4 of 10	0 of 10 after 2 months 4 of 10 after 2 years
Choi et al. (2024) [29]	VAS	8.3 (range 6 to 10)	Group 1: persistent pain after 3 months conservative treatment (*n* = 14) 6.7 (range 5 to 9) Group 2: relieved pain after 3 months conservative treatment (*n* = 13) 2.3 (range 1 to 5)

**Table 5:** Functional outcomes before and after treatment in patients with sesamoid-related disorders, measured using the Foot and Ankle Outcome Score (FAOS) and the Foot and Ankle Ability Measure (FAAM) subscales.

Study	Scale	Functionality Before Treatment	Functionality After Treatment
Shimozono et al. (2022) [30]	FAOS	Symptoms 64 (7.4) Pain 56.1 (11.4) Daily activities 68.9 (8.6) Sports activities 36.8 (9.4) Quality of life 30.5 (8.2) **Overall 51.3 (5.6)**	Symptoms 90.8 (7.5) Pain 83.8 (17.4) Daily activities 92.8 (7.3) Sport activities 72.5 (20.1) Quality of life 64.6 (22.7) **Overall 81.4 (13.4)**
Choi et al. (2024) [29]	FAAM-ADL	14.7 (12 to 21)	Group 1: 14.3 (11 to 22) Group 2: 26.6 (23 to 32)
	FAAM-Sports	51.9 (42 to 63)	Group 1: 52.1 (42 to 64) Group 2: 73.4 (68 to 79)

**Table 6:** Return-to-activity outcomes following various treatment modalities for sesamoid-related disorders. Reported endpoints include time to return, ability to resume prior level of sports or daily activity, and persistence of musculoskeletal pain (MSP).

Study	Return to Activity
Axe and Ray (1988) [28]	Five varsity athletes: three patients had immediate and almost complete relief of symptoms with orthoses, one patient had a gradual resolution of symptoms over 4 weeks, one required 6 weeks for pain resolution. Without orthoses, three had persistent symptoms during basketball and one during activities of daily living. They refused surgery. Two recreational runners remained symptomatic without the orthoses and used them prophylactically. Without orthoses one patient had persistent symptoms during aerobics and the other while wearing heels. They refused surgery. One musician became immediately asymptomatic with the orthoses and then without orthoses. The remaining two patients underwent the surgical excision of a sesamoid. In particular, the professional dancer returned to ballet after 3 months and the jogger to extended distances after 3 months (post surgery).
Frey and Koehle (2013) [26]	After 3 months, the patient was able to return to running, and 4 months later she completed a 10 km race in 75 min with minimal ongoing discomfort.
Shin et al. (2013) [24]	The following day the patient reported no pain and went shopping.
Thompson et al. (2017) [25]	The patient returned to playing tennis 6 weeks after completing the treatment and undergoing a 12-week sport-specific rehabilitation programme.
Shimozono et al. (2022) [30]	Eight of eleven (73%) patients that were involved in sports prior to the CBMA injection returned to play, with seven successfully returning to preinjury level status (three professional, one college, and three recreational levels).
Callahan et al. (2024) [20]	After 3 months, the patient returned to regular ballet auditions.
Choi et al. (2024) [29]	Group 1 after 6 months (*n* = 12): Able to reach prior level of sports activity without pain: 0 (0%); Able to participate in sports, but experiencing mild post-sports MSP: 3 (25%); Able to perform activities of daily living, but experiencing MSP during sports activities and unable to engage in sports: 5 (41.7%); Experiencing residual MSP during activities of daily living: 4 (33.3%). Group 2 after 6 months (*n* = 11): Able to reach prior level of sports activity without pain: 5 (45.4%); Able to participate in sports, but experiencing mild post-sports MSP: 4 (36.4%); Able to perform activities of daily living, but experiencing MSP during sports activities and unable to engage in sports: 2 (18.2%); Experiencing residual MSP during activities of daily living: 0 (0%).

**Table 7:** Quality assessment of the studies included in the present systematic review.

Study	Quality Items									
	Q1	Q2	Q3	Q4	Q5	Q6	Q7	Q8	Q9	Q10
	Case Series Studies									
Axe and Ray (1988) [28]	Yes	Yes	Yes	No	Yes	Yes	Yes	Yes	Yes	N/A
Aşkın et al. (2017) [27]	N/A	Yes	Yes	No	Yes	Yes	Yes	Yes	N/A	N/A
Shimozono et al. (2022) [30]	Yes	Yes	Yes	Yes	No	Yes	Yes	Yes	Yes	N/A
Choi et al. (2024) [29]	Yes	Yes	Yes	Yes	No	Yes	Yes	Yes	Yes	N/A
	Case Reports									
Frey and Koehle (2013) [26]	Yes	Yes	Yes	Yes	Yes	Yes	N/A	Yes	–	–
Shin et al. (2013) [24]	Yes	Yes	es	Yes	Yes	Yes	N/A	Yes	–	–
Kumar et al. (2015) [22]	Yes	Yes	Yes	Yes	Yes	N/A	N/A	Yes	–	–
Thompson et al. (2017) [25]	Yes	Yes	Yes	Yes	Yes	Yes	N/A	Yes	–	–
Benslima et al. (2023) [21]	Yes	Yes	Yes	Yes	Yes	Yes	N/A	Yes	–	–
Callahan et al. (2024) [20]	Yes	Yes	Yes	Yes	Yes	Yes	N/A	Yes	–	–
Maganinho et al. (2024) [23]	Yes	Yes	Yes	Yes	Yes	Yes	N/A	Yes	–	–

## Data Availability

The original contributions presented in the study are included in the article.

## Abbreviations

BMI	Body Mass Index
CBMA	Concentrated Bone Marrow Aspirate
FAAM	Foot And Ankle Ability Measure
FAOS	Foot And Ankle Outcome Score
JBI	Joanna Briggs Institute
MTP	Metatarsophalangeal
NSAIDs	Nonsteroidal Anti-Inflammatory Drugs
NRS	Numeric Rating Scale
NWB	Non-Weight-Bearing
PAC	Passive Axial Compression
PRISMA	Preferred Reporting Items for Systematic Reviews and Meta-Analyses
PRP	Platelet-Rich Plasma
RCT	Randomised Controlled Studies
VAS	Visual Analogue Scale
